# New Signatures of Bio-Molecular Complexity in the Hypervelocity Impact Ejecta of Icy Moon Analogues

**DOI:** 10.3390/life12040508

**Published:** 2022-03-30

**Authors:** Surendra V. Singh, Haritha Dilip, Jaya K. Meka, Vijay Thiruvenkatam, Vishakantaiah Jayaram, Mariyappan Muruganantham, Vijayan Sivaprahasam, Balabhadrapatruni N. Rajasekhar, Anil Bhardwaj, Nigel J. Mason, Mark J. Burchell, Bhalamurugan Sivaraman

**Affiliations:** 1 Physical Research Laboratory, Ahmedabad 380009, India; jayakrishna@prl.res.in (J.K.M.); vmmuruga@gmail.com (M.M.); vijayansiva@gmail.com (V.S.); bhardwaj_spl@yahoo.com (A.B.); 2 Indian Institute of Technology, Gandhinagar 382355, India; d_haritha@iitgn.ac.in (H.D.); vijay@iitgn.ac.in (V.T.); 3 Indian Institute of Science, Bangalore 560012, India; drjayaramv@gmail.com; 4 Bhabha Atomic Research Centre, Mumbai 400085, India; bnrs@rrcat.gov.in; 5 School of Physical Sciences, University of Kent, Canterbury CT2 7NZ, UK; m.j.burchell@kent.ac.uk

**Keywords:** amino acids, polypeptides, impact ejecta, icy moons, astrobiology

## Abstract

Impact delivery of prebiotic compounds to the early Earth from an impacting comet is considered to be one of the possible ways by which prebiotic molecules arrived on the Earth. Given the ubiquity of impact features observed on all planetary bodies, bolide impacts may be a common source of organics on other planetary bodies both in our own and other solar systems. Biomolecules such as amino acids have been detected on comets and are known to be synthesized due to impact-induced shock processing. Here we report the results of a set of hypervelocity impact experiments where we shocked icy mixtures of amino acids mimicking the icy surface of planetary bodies with high-speed projectiles using a two-stage light gas gun and analyzed the ejecta material after impact. Electron microscopic observations of the ejecta have shown the presence of macroscale structures with long polypeptide chains revealed from LCMS analysis. These results suggest a pathway in which impact on cometary ices containing building blocks of life can lead to the synthesis of material architectures that could have played a role in the emergence of life on the Earth and which may be applied to other planetary bodies as well.

## 1. Introduction

Extraterrestrial impacts are thought to be one of the potential ways that provided the necessary chemical ingredients to the early Earth and thus played a major role in the origin of life [1,2]. Indeed, the detection of biomolecules in meteorite samples has confirmed the abiotic origin of such molecules [3], with recent measurements confirming high abundances of amino acids in meteorites [4]. The detection of the simplest amino acid glycine from the sample return of the NASA Stardust mission to comet 81P/Wild 2 [5] and confirmation by the ROSINA mass spectrometer of its presence in Comet 67P during the Rosetta mission [6], has demonstrated that larger bodies in space contain important organics. Recent reports have suggested a mechanism of glycine formation in the interstellar medium without the presence of any energetic sources [7]. The role of meteorites has also been studied in terms of assisting the synthesis of nucleosides and nucleotides [8,9]. Thus exogenous sources could have provided a potential amount of biologically important organics to the early Earth and thus could have been a major source of the Earth’s organic budget.

However, given the catastrophic nature of large-scale impact and its related events, the survival of organics in such extreme environments of high temperature and pressure remains uncertain. In addition, impact bombardment of comets and asteroids cause significant damage to the planetary surface upon impact, resulting in the formation of craters, melts, shocked surface materials and vapours [10]. Many impact craters have been observed on the surface of various planetary bodies, a record of impact history that shows impacts are widespread in the solar system and play a significant role in solar system formation and evolution. Impact induced shock provides a sharp increase in pressure and temperature due to sudden shock compression and subsequent cooling due to expansion within a very short time scale which has the potential for driving chemical reactions [11,12,13]. Thus, the role of impact bombardments in prebiotic chemistry and the emergence of life on Earth must be explored.

Many experimental and theoretical simulations are available in the literature that discusses the role of impact processes in prebiotic chemistry and the origin of life. The famous Urey-Miller experiment [14] on the synthesis of amino acids in simulated Earth’s atmosphere (using a simple mixture of gases), triggered the search for the synthesis and survivability of amino acids and other biomolecules. Many experiments have been performed to investigate the survivability of amino acids and other organics in impact-shock environments, many of which have confirmed their survivability and determined the survival rates, e.g., [11,15,16,17,18,19,20]. Not only do these biomolecules survive, but shock environments can assist the synthesis of biomolecules. For example, Bar-Nun et al. [21] reported the shock synthesis of amino acids in a simulated Earth’s primitive atmosphere by shock processing of simple gases using a shock tube. Further experimental investigations reported the formation of many amino acids by simulating the impact on icy bodies of the solar system [22] and impact into an early ocean [23,24,25]. Nucleobases are also known to be a product of processes related to impact events [24,26]. Ferus et al. [27,28,29,30] recently reported the synthesis of nucleobases and amino acids starting from formaldehyde, mimicking high-velocity asteroidal impact using a high-intensity laser. Di- and tri-peptides are also synthesized by simulating impacts into icy bodies starting from the amino acid [31,32]. Studies have also shown chemical complexity in the interstellar environment as a result of shock compression resulting from icy dust collision [33]. All of these studies have confirmed the availability of a vast number of biologically important molecules in prebiotic Earth and other planetary bodies due to impact bombardment and related events.

However, given the history of impact bombardment on early Earth, the study of the effect of impact events and their influence on complex molecular synthesis must be explored. In previous investigations, we have shown the formation of complex macroscale structures due to the shock processing of amino acids at extreme temperatures [34]. In this study, we report finding complex organized structures comprised of polypeptides, synthesized in the ejecta material after a projectile is fired on a target containing amino acid embedded in water ice. We started the experiments with the simplest amino acid glycine and then glutamine, which has an amide group in the side chain. Though glycine was perhaps the major product in a prebiotic scenario, the significance of other amino acids cannot be neglected [35]. Further experiments were performed with the mixture of the two amino acids, which is a more realistic scenario for the prebiotic chemistry where interaction among different amino acids occurred [36,37]. The concentration of amino acids used in the present investigation is unusually high compared to the meteoritic abundance or any plausible prebiotic scenario. It is essential to mention that the reactions in simulated prebiotic conditions proceed in an uncontrolled way and result in unwanted products [38], and desirable products must be within the detection limit [39]. So the present study requires a higher concentration of amino acids. In our future studies, we will be performing experiments with realistic concentrations of amino acids mimicking meteoritic composition.

## 2. Materials and Methods

The hypervelocity impact experiments were performed utilizing a two-stage light gas gun facility at the University of Kent. The instrument is capable of firing projectiles of size 0.1 mm to 3.0 mm diameters over a velocity range of 0.3 to 7.5 km s^−1^. The detailed instrument parameters and capabilities can be found in Burchell et al. [40] and Hibbert et al. [41]. In the present investigations, we impacted a spherical projectile (stainless steel 420), 1 mm in diameter, at a velocity of approximately 5 km s^−1^ on amino acid-water ice targets. The amino acids of purity >99% were procured from Sigma-Aldrich. Single amino acids glycine and glutamine, as well as a mixture of the two amino acids mixed in an equal weight ratio to a total weight of 3.5 g were dissolved entirely in 150 mL HPLC grade water using a magnetic stirrer in a glass beaker and then cooled to 1.5 °C. In a separate steel beaker, water ice is prepared frozen at −20 °C. The solution of amino acid-water was then poured into the beaker containing the water ice, and this whole mixture is cooled to 140 K in a freezer. These steps are detailed in Supplementary Figure S1. The targets were kept in the freezer for almost 15–20 h and removed from the freezer just before the firing and mounted in the target chamber (Supplementary Figures S2 and S3). The target chamber walls were covered with aluminium foil to collect the ejecta from the target after the impact process. The target chamber was evacuated to 50 mbar before the firing of the gun. After the firing was done and the target chamber returned to atmospheric pressure, the ejected materials from the target were left on the aluminium foil in the chamber to dry out entirely at room temperature and then collected for further analysis. The peak shock pressure for each experiment is estimated using planar impact approximation [42]. This method assumes a linear shock-wave speed relationship for each material in the impact (water ice and stainless steel bullet) [43,44]. The material-specific empirical constants are obtained from Melosh [42]. The experimental parameters for different experiments are shown in Table 1. A target with only water ice, when impacted, did not show any residue on the aluminium foil, while an amino acid-water ice target resulted in a white residue left on the aluminium foil as shown in Supplementary Figure S4. The foils containing the residues were sealed in boxes and transported to PRL Ahmedabad. These residues were then further analyzed using SEM, TEM and LCMS. The detailed methodology for SEM, TEM and LCMS are provided in the Supplementary Material (Figure S5).

## 3. Results

### 3.1. Morphological Analysis of Ejecta

**Figure 1:**
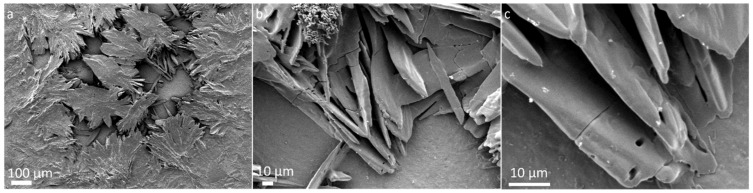
SEM micrographs of glycine ejecta after impact show (**a**) large clumped structures (**b**,**c**) more magnified images reveal sharper structures.

Motivated by our previous results revealing fascinating structures produced by shock processing of amino acids [34], we explored the intricate details of surface morphology and structures found in the ejecta using a field emission scanning electron microscope (FESEM). FESEM observations revealed remarkable morphological features that are present in the ejecta. Ejecta from different targets yielded unique morphological characteristics. Glycine ejecta showed the formation of large clumps, hundreds of microns in size, as shown in Figure 1a. More magnified images showed various sharp structures clumped together, as shown in Figure 1b,c. Ejecta from different places of foil showed similar results.

**Figure 2:**
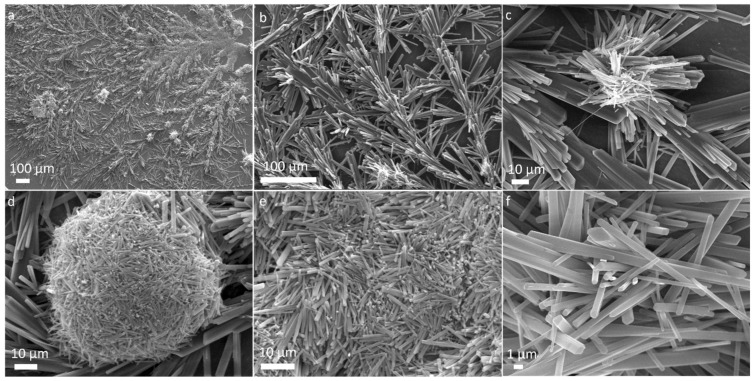
SEM micrographs of glutamine ejecta show (**a**) large dendritic structures (**b**,**c**) more magnified images show branching features (**d**) spherical assembly of nanorods (**e**,**f**) more magnified images shows rod-like structures of various length.

When observed using FESEM, ejecta from shocked glutamine revealed an entirely different pattern. Large dendritic structures with various branching features were observed with an upward orientation from nucleation points ranging in size up to few millimetres, as shown in Figure 2a. More magnified structures of these branching features are shown in Figure 2b,c. These dendritic structures have a resemblance with dendritic structures observed in the self-assembly of peptides [45]. Apart from dendritic structures, a spherical assembly of rods were also found as shown in Figure 2d. More magnified images show these rods to have geometrical shapes and to be of varying size, typically tens of micrometres (Figure 2e,f). Images were from multiple sites on the aluminium foils were analyzed in the SEM and similar structures were found.

**Figure 3:**
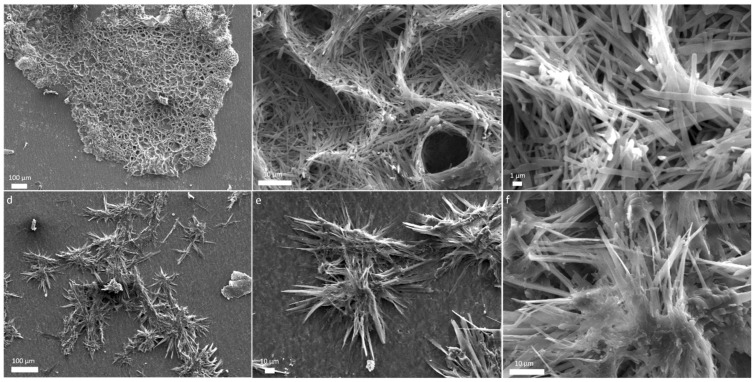
SEM micrographs of glycine-glutamine ejecta show (**a**) an array of large aggregates (**b**,**c**) magnified images show organized structures made of micro-ribbons, (**d**) assembly of needle-shaped fibers, and (**e**,**f**) more magnified microstructures.

Ejecta from the mixture of glycine and glutamine revealed large aggregates a few millimetres in size (Figure 3a). More magnified images revealed nest-like intertwined organized structures formed by association of thin ribbons tens of micrometres in length. Sample collected from another site on the foil, for the same mixture, we found entirely different structures as shown in Figure 3d. An array of needle-shaped fibers 100 micrometres in size were observed oriented in various directions. More magnified microstructures are shown in Figure 3e,f. The difference in various structures that are observed in different amino acids may be due to the difference in the side chains of amino acids and the various interactions responsible for driving such assembly [46]. Amino acids and peptides are known to form self-assembled nanostructures [46,47]; however, formation of complex structures at such extreme conditions is rarely reported.

**Figure 4:**
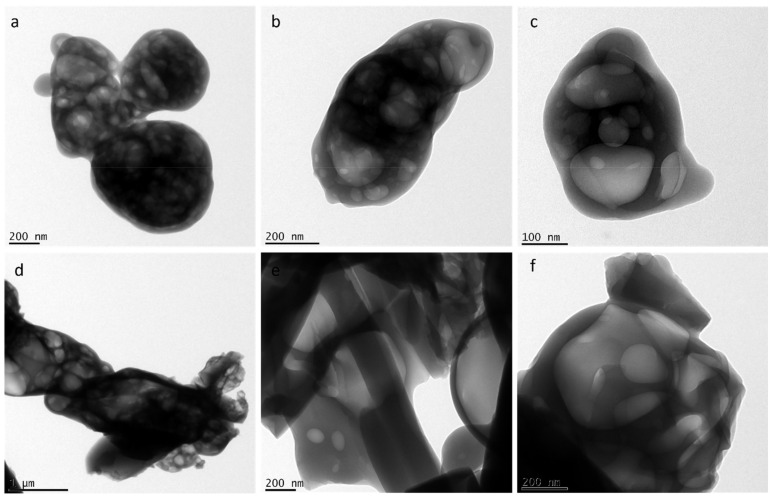
TEM micrographs of (**a**–**c**) glycine ejecta and (**d**–**f**) glycine–glutamine ejecta.

Ejecta residues were also subjected to a high resolution transmission electron microscope (HRTEM). HRTEM micrographs of glycine and glycine–glutamine samples are shown in Figure 4. HRTEM observation of both the samples showed a multi-layered porous structure with membrane-like appearances at the nanometre scale.

During target preparation for experiments, while dissolving the amino acid in water, control samples were prepared by drop casting the amino acid-water solution on aluminium foil. These samples, when analyzed in the FESEM did not show any such organized structure formation as shown in Supplementary Figure S6. This confirms that the organized structures that are observed in the ejecta materials results from impact induced processes.

### 3.2. Mass Spectrometry of Ejecta Materials

**Figure 5:**
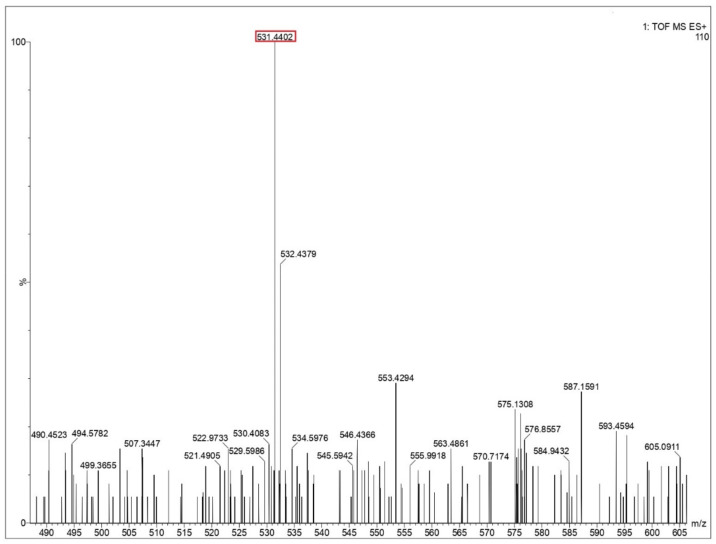
Mass spectra of glycine residue obtained after impact. Identified peptide peak (M^+^) is shown in the red box corresponds to 531.4402 matches with calculated value 531.466 (peptide sequence of 9 G’s). Y-axis shows relative abundance.

**Figure 6:**
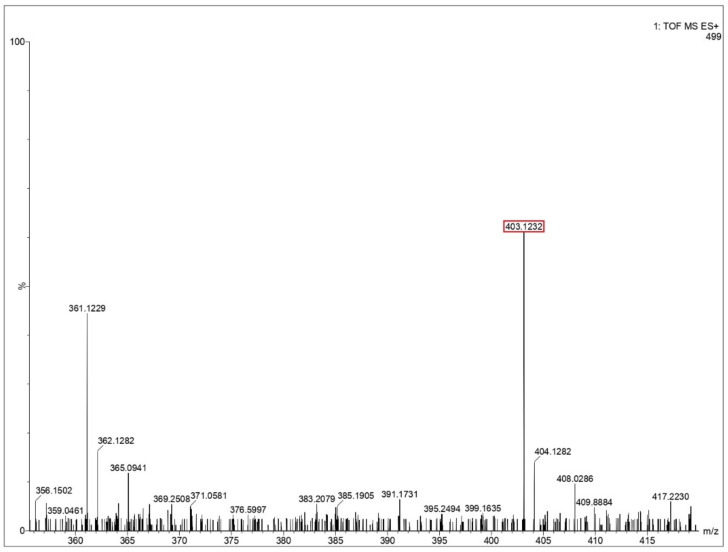
Mass spectra of glutamine residue obtained after impact. Identified peptide peak (MH^+^) is shown in red box corresponds to 403.1232 matches with calculated value 402.391 (peptide sequence—3 Q’s). Y-axis shoes relative abundance.

Ejecta residue showing the complex macroscale structures were analyzed using liquid chromatography mass spectrometry (LCMS). The appropriate steps taken for LCMS analysis are provided in the Supplementary File. An approach to LCMS analysis as a tool to obtain chemical composition and fragmentation details in macromolecular complexes has been well reported in literature [48]. The major advantage in deploying LCMS in the present study includes the improved characterization of complex samples with an enhanced resolution, which increases the analytical value of the samples while simultaneously combining its optical and mass data. The chances of occurrence of false positives can be ruled out since the LCMS spectra of the blank solution (Milli-Q water), containing all the components other than the respective amino acids under study, have also been analyzed at the same conditions. A comparative study between the blank solution and the test samples showed that the reported peaks are characteristic only to the test samples and not the blank. The mass spectra from glycine, glutamine and glycine–glutamine mixtures are shown in Figure 5 and Figure 6, and Supplementary Figures S7–S14. Long polypeptide chains were identified in the ejecta by comparing the theoretical value of peptides calculated from the peptide mass calculator [49] by putting in the constituent amino acids. These values for different peptides are listed in Table 2 for all three samples. LCMS analysis of ejecta from glycine showed that various polypeptides were synthesized as a result of the impact. The different peptide sequence which could be identified corresponds to the sequence of three, five, nine and twelve glycines, as shown in Figure 5 and Supplementary Figures S7–S9. Ejecta from the glutamine sample also showed long polypeptide chains, as shown in Figure 6 and Supplementary Figures S10 and S11. The identified peptide sequence corresponds to the sequence of two, three and five amino acids. Further, the mixture of glycine and glutamine also showed synthesis of long polypeptide chains with various combinations of two amino acids, as shown in Supplementary Figures S12–S14 and the corresponding sequences are listed in Table 2. These results prove that long polypeptide chains are synthesized in the ejecta as a result of the impact on amino acids. Long polypeptide chains up to sequence of twelve amino acids as observed in case of glycine is remarkable and has never been reported before. Previous studies have reported shorter peptides using gas gun experiments [31,32] and ball milling techniques [50] simulating extraterrestrial impacts.

There may be other products present in the ejecta that could not be found among the different products identified by LCMS analysis. Also, as shown in the Supplementary Figure S4, the ejecta on the aluminium foils is scattered on various places in an area of aluminium foil of about one-meter square. Extensive analysis will be needed to scan the samples from multiple positions of foil and identify those undetermined products.

## 4. Discussion

The hypervelocity impact experiments on amino acid targets performed in the present study at impact speed of approximately 5 km s^−1^, creating a peak pressure of approximately 30 GPa. The typical impact speed observed in natural impact events cover a wide range of values and may be significantly different from those achieved in the laboratory and are almost impossible to simulate [44]. The rational for the choice of impact speed in the present study is to maximally mimic the physical conditions observed in natural impact events. There are several plausible scenarios where the pressure values in natural impacts can be reduced significantly due to atmospheric drag, airburst in the atmosphere, fragmentation and oblique impacts [31,44,51,52]. Further, in a single impact, pressure values can differ significantly as pressures are distributed heterogeneously around various points of an impacting object [52]. Thus, a range of pressure values are achieved in natural impact events and we have tried to simulate a part of such values in our experiments.

The results obtained from SEM and LCMS analysis demonstrate that amino acids reacted strongly within a short time scale to form organized structures and long polypeptide chains due to the high pressures and temperatures incurred during the impact and in post-impact relaxation. The individual chemical composition of each structure is difficult to determine; however, their similarity with known structures obtained from various peptide self-assembly indicates that these structures can possibly arise as a result of the assembly of various polypeptides synthesized upon impact.

Previous studies have suggested that extraterrestrial impacts can contribute to the synthesis of peptides by impact-driven processes on prebiotic Earth and other planetary bodies [11,31,32]; however, the study of ejecta material with such complex structure has not been previously reported. The extant biology is characterized by an interconnecting network of biopolymers such as proteins, lipids, nucleic acids with peptides playing a central role in mediating these cellular networks because of their unique architecture and functionality [53]. Thus, the abiotic synthesis of peptides is an important step in the prebiotic chemistry that led to the emergence of life on the primitive Earth. Various scenarios have been suggested on the synthesis of peptides on the prebiotic Earth, which include synthesis in hydrothermal vents and dehydrating condition under dry and wet cycles [54]; synthesis triggers by activating agents [55,56]; presence of mineral catalysis [57,58], high energy protons and UV irradiation [59,60]; volcanic environment [37] and mechanochemical synthesis [50]. However, long polypeptide chains, as found in the present investigation, has not been reported before. The result that the various sequence of different amino acids can combine together to form long polypeptide chain due to impact process is thus interesting.

The first step among the many challenges in the origin of life is to look for a simple structure formed by self-assembly, which is sufficiently complex to assume the properties of biological system and to determine how these processes happened on the early Earth environment. The origin of life conundrum requires the availability of building blocks of life that are compatible with the environments that may have existed on early Earth and a further increase in molecular complexity via self-organization processes [61,62,63]. However, a great challenge remains on the design of such structural architecture from basic ingredients in the plausible prebiotic environment [64,65,66,67]. The role of synthetic microstructures in the origin of life has been discussed previously, and various prebiotic conditions have been suggested for the synthesis of such structures, which includes quenched spark discharge experiments [68], formation of lipid-like structures [69,70,71] and tubular structures in mineral surfaces [72]. In particular, various protocellular structures were obtained from polypeptide formation from four amino acids simulating in a hydration-dehydration cycle of the tidal pool [73], and microspheres were synthesized from simple molecules under the simulated condition of prebiotic times [74]. Further, lipid-like self-assembling peptides were synthesized from amino acids forming tubular and vesicle structures [75]. However, the formation of complex organized structures from the building blocks of life as a result of impact processes, as revealed in the present investigation, provides a new and significant route towards the origin of life. Many significant challenges in this field are yet to be explored. The formation of complex architectures revealed in the present investigations are the first step among the many challenges in this path.

## 5. Conclusions

Thus, our results demonstrate that complex macroscale structures were synthesized in the impact and can be observed in the ejecta. Mass spectrometric analysis shows the presence of many polypeptides in the ejecta. These results provide a pathway for the building blocks of life to evolve into complex organized structures, which has implications for the origin of life. This is the first report on the formation of long polypeptide chains to be synthesized under plausible prebiotic conditions with a combination of the same and two different amino acids. As impacts are widespread in the solar system, these results could be applied to the icy bodies of the solar system, such as the icy satellites of Jupiter and Saturn, to understand the chemical composition and evolution of icy surfaces. It is expected that these icy bodies must have been supplied with a significant amount of organics as a result of impact events and thus could possibly have synthesized peptides as well due to impact-induced shock processes. If we are looking for the signature of precursors of life on icy bodies, the ejecta materials around the craters will be an ideal place to search for them.

## Figures and Tables

**Table 1:** Experimental parameters for each experiment.

Target	Impact Velocity (km s^−1^) Accurate to ±1%	Peak Shock Pressure (GPa)	Ejecta
Pure water ice	4.80	29.0	No residue
Glycine	5.09	32.1	White ejecta
Glutamine	4.66	27.6	White ejecta
Glycine-Glutamine	4.77	28.7	White ejecta

**Table 2:** Identified peptide mass number and retention time obtained from the mass spectra (Figure 5 and Figure 6 and Supplementary Figures S7–S14) corresponding to the calculated average mass and sequence of peptides obtained from the Peptide Synthetics peptide mass calculator [49].

Peak Value (*m*/*z*)	Retention Time (min)	Sequence	Calculated Value
Glycine (G)			
189.0200	0.553	GGG	189.155
246.2485	4.963	GGGG	246.207
531.4402	13.094	GGGGGGGGG	531.466
702.9087	17.011	GGGGGGGGGGGG	702.621
Glutamine (Q)			
275.0708	2.625	QQ	274.261
403.1232	9.447	QQQ	402.391
659.3535	15.439	QQQQQ	658.652
Glycine (G)-Glutamine (Q)			
204.0777	8.389	1 G and 1 Q	203.182
332.2456	8.389	1 G and 2 Q’s	331.312
517.2334	9.675	2 G’s and 3 Q’s	516.495
773.6675	10.981	2 G’s and 5 Q’s	772.756

## Data Availability

Samples of the compounds that support these findings are available from the corresponding authors upon request.

## Supplementary Materials

The following supporting information can be downloaded at: https://www.mdpi.com/article/10.3390/life12040508/s1, Figures S1–S5: Experimental details; Figures S6 and S7: SEM micrograph; Figures S7–S14: LCMS data.
