# Supplementary material for: New Signatures of Bio-Molecular Complexity in the Hypervelocity Impact Ejecta of Icy Moon Analogues
Source: Life (Basel). 2022 Mar 30;12(4):508. doi: 10.3390/life12040508 (PMC9026792; doi:10.3390/life12040508)
Supplement: Supplementary file 1 [file life-12-00508-s001.zip › life-1610361-supplementary.pdf]

## Supplementary Materials

## Experimental details

### Preparation of target

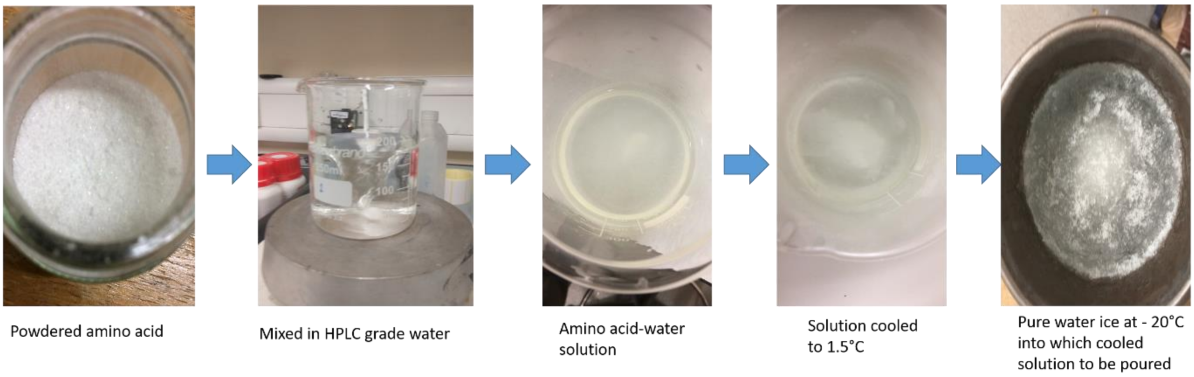


Figure S1. Step taken for the preparation of amino acid-water ice target

### Target and chamber

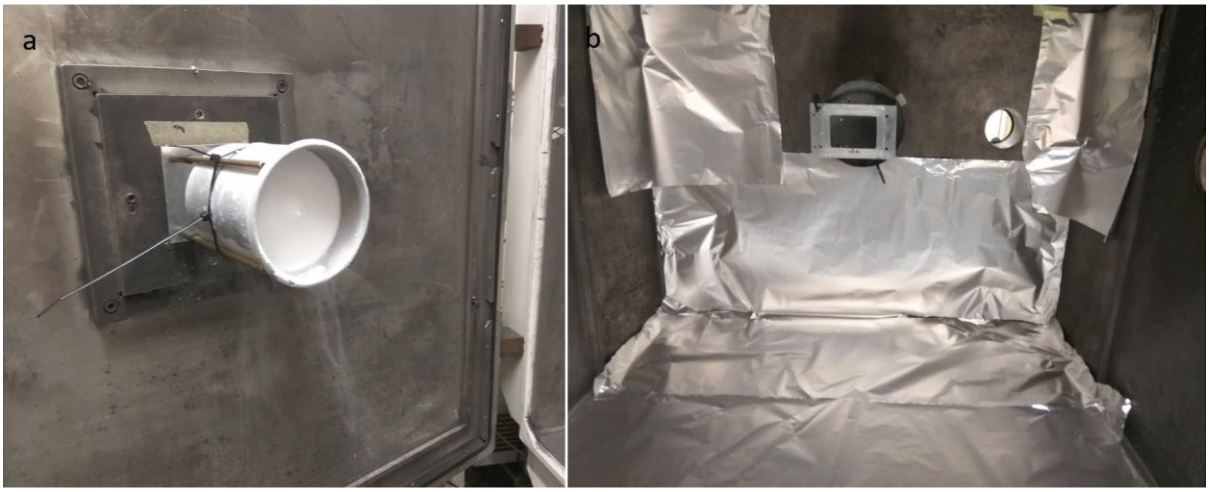


Figure S2. Amino acid-water target frozen at 140 K mounted at the back door of the chamber and chamber walls covered with aluminium foil

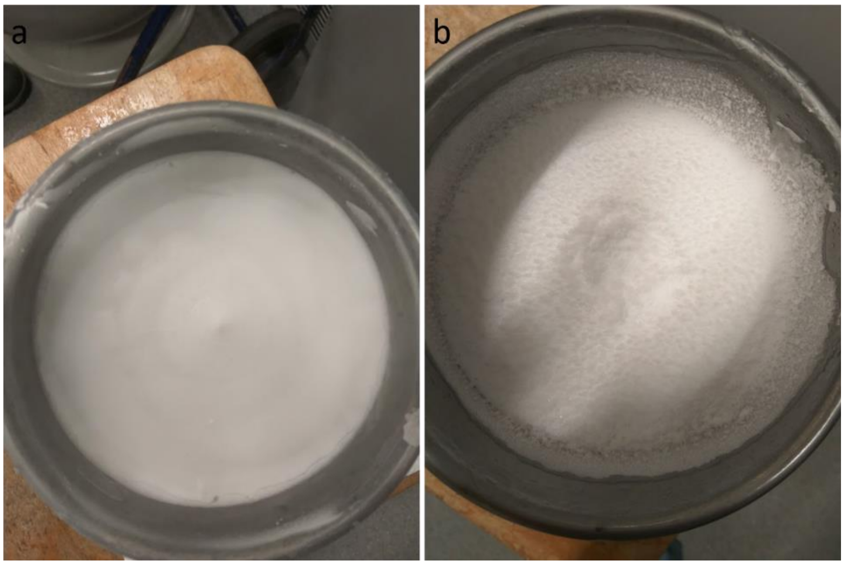


Figure S3. Target (a) before and (b) after impact, a small crater formed after impact.

**Ejecta**

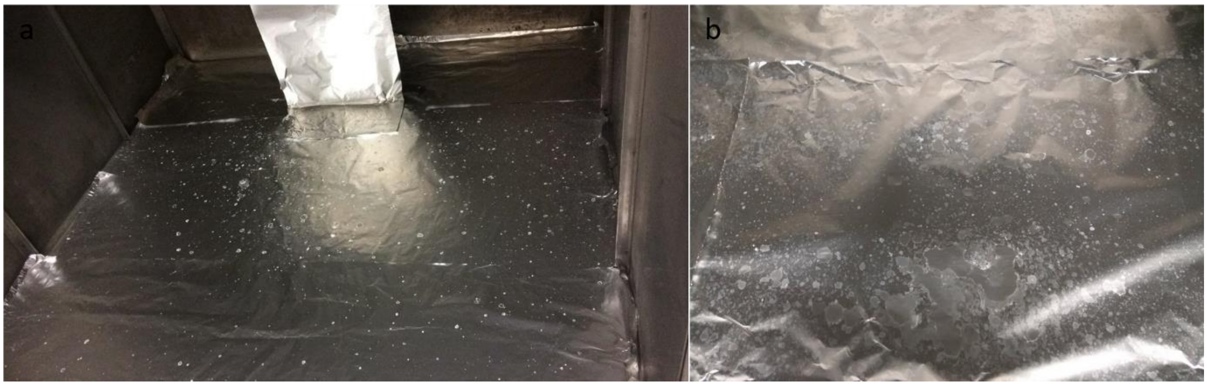


Figure S4. Ejected material after impact left out to dry at room temperature in the chamber and then collected for further analysis.

**SEM analysis**

Aluminium foils containing the ejecta materials were cut into small pieces and mounted on SEM sample stubs with help of conductive carbon tape and the top surface of sample stub coated with gold for 50 seconds to make the surface conductive. Samples were subjected to a SEM at an operating voltage of 5 kV and micrographs were obtained at magnification of 100 to 10000 using JSM7600F (Jeol) instrument at IIT Gandhinagar.

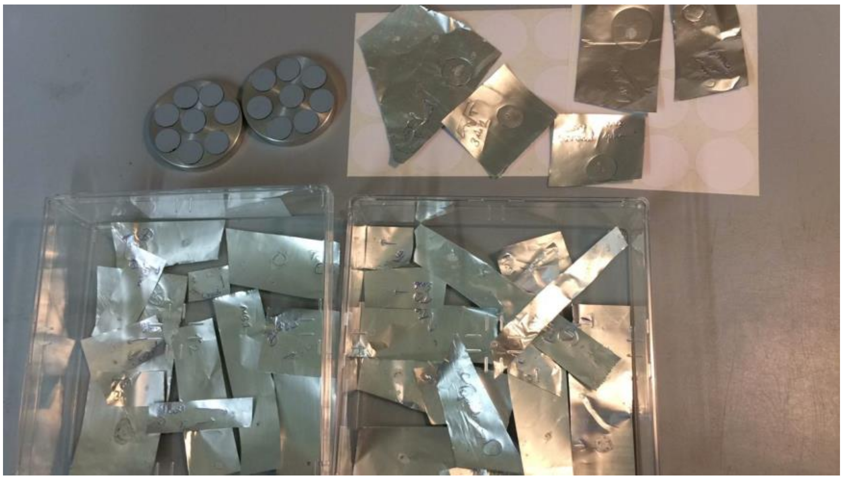

Figure S5. Aluminium foils containing the ejecta subjected to SEM

**Control**

Amino acids dissolved in water and then drop casted on aluminium foil and analysed using SEM. SEM micrographs of glycine and glycine-glutamine sample is shown below.

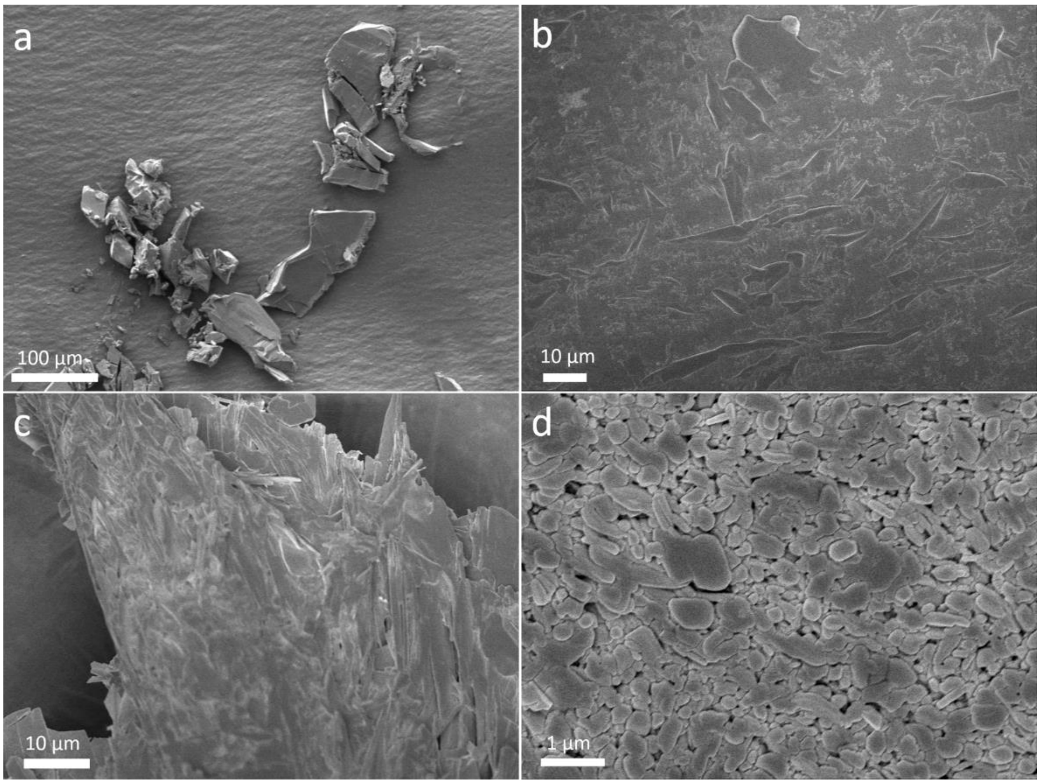


Figure S6. SEM micrographs of control showing large particles and aggregates

**TEM analysis**

High resolution transmission electron microscope (HRTEM) analysis was performed to investigate the internal structure ejecta residue at higher magnification than just SEM. A small quantity of sample was placed in 1.5 ml of acetone solvent and then sonicated for 10-20 min. If the solution looks dark the sample may agglomerate and it is not suitable to get good HRTEM images. Acetone is further added to make the solution more dilute and a micro pipette used to collect the suspended particles from the dilute solution and a drop of this solution was dispersed on 200 mesh carbon coated copper grid. The sample was kept in vacuum desiccators before loading into the microscope**.** HRTEM studies were performed utilizing HRTEM (Model: FEI –TECNAI G $^{2}$ -20 TWIN), at an operating voltage of 200 kV and magnification of approximately 20000×.

**LCMS Sample preparation and analysis**

Residue from the aluminium foil were collected and analysed in the Waters Acquity UPLC-SYNAPT G2-S MS system. The ACQUITY UPLC H-Class performs both UPLC and HPLC functions, and can achieve high resolution, speed and sensitivity in liquid chromatography analysis, compared to conventional systems. The SYNAPT G2-MS is hybrid, quadrupole/orthogonal acceleration, time of flight mass spectrometer controlled by MassLynx software.

The tiny amount of residue was dissolved in HPLC grade water. 100 µL of this sample was dissolved in 1 mL HPLC grade methanol to prepare a 100 µL/mL solution. The sample was injected at a flow-rate of 0.05 ml/min into UPLC with an injection volume of 5 µL. The sample components were separated using a waters ACQUITY UPLC BEH C18 1.7 µm column, with dimensions 2.1×100 mm. The column, sample and room temperatures were maintained at 30°C, 20°C and 22°C respectively. The mobile phase consisted of (A) water containing 0.1% formic acid and (B) acetonitrile containing 0.1% formic acid. The gradient for mobile phase started from 95% solvent A and 5% solvent B, decreasing linearly to 0% solvent A and 100% B in 20 minutes, at a flow-rate of 0.4 mL/min. The sample was analysed by using a Lockspray source (Leucine, flowrate 0.3 ml/min), with ESI ionization and positive ion modes. The TOF mass range for the instruments is from m/z 50-2000. The analysis and assigning of peaks were carried out using the MassLynx software.

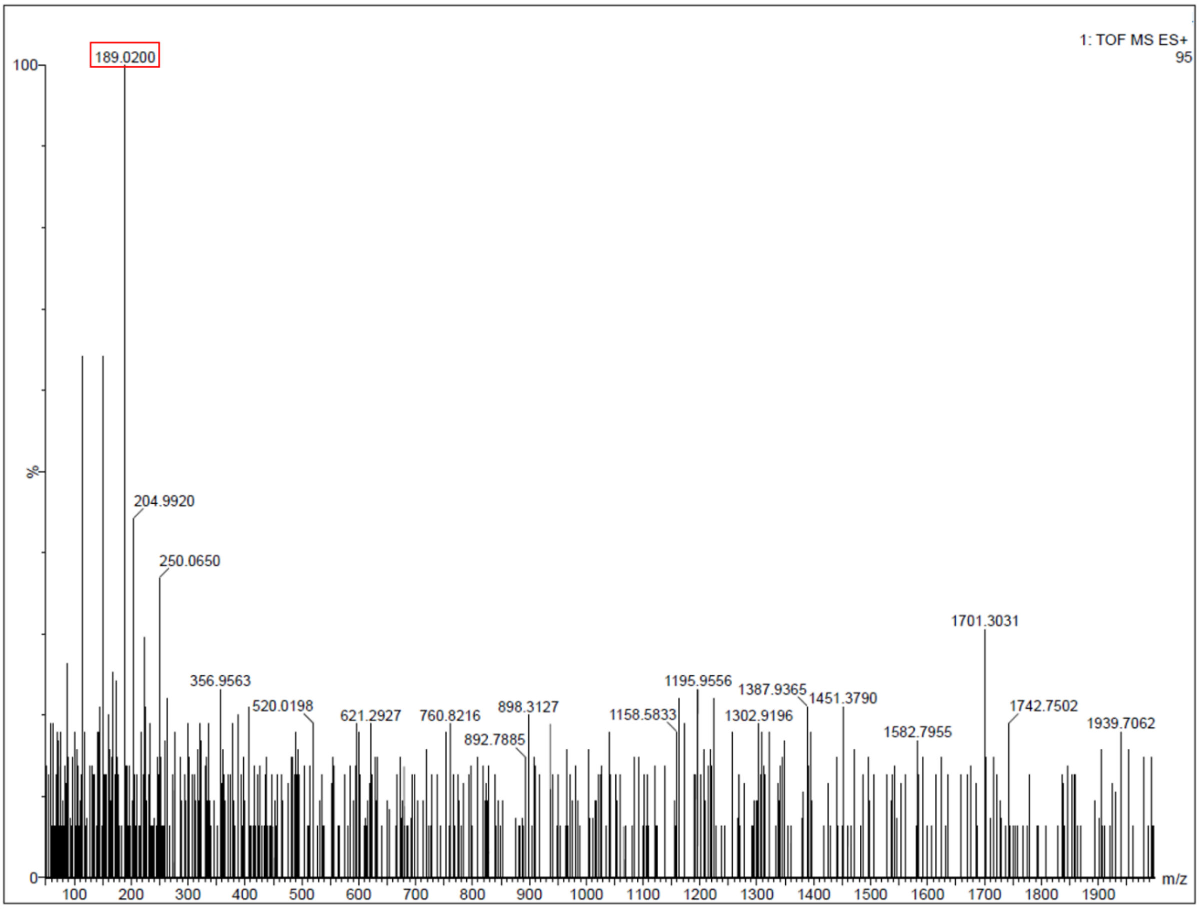


**Figure S7.** Mass spectra of glycine residue obtained after impact. Identified peptide peak ($M^+$) is shown in red box corresponds to 189.0200 matches with calculated value 189.155 (GGG)

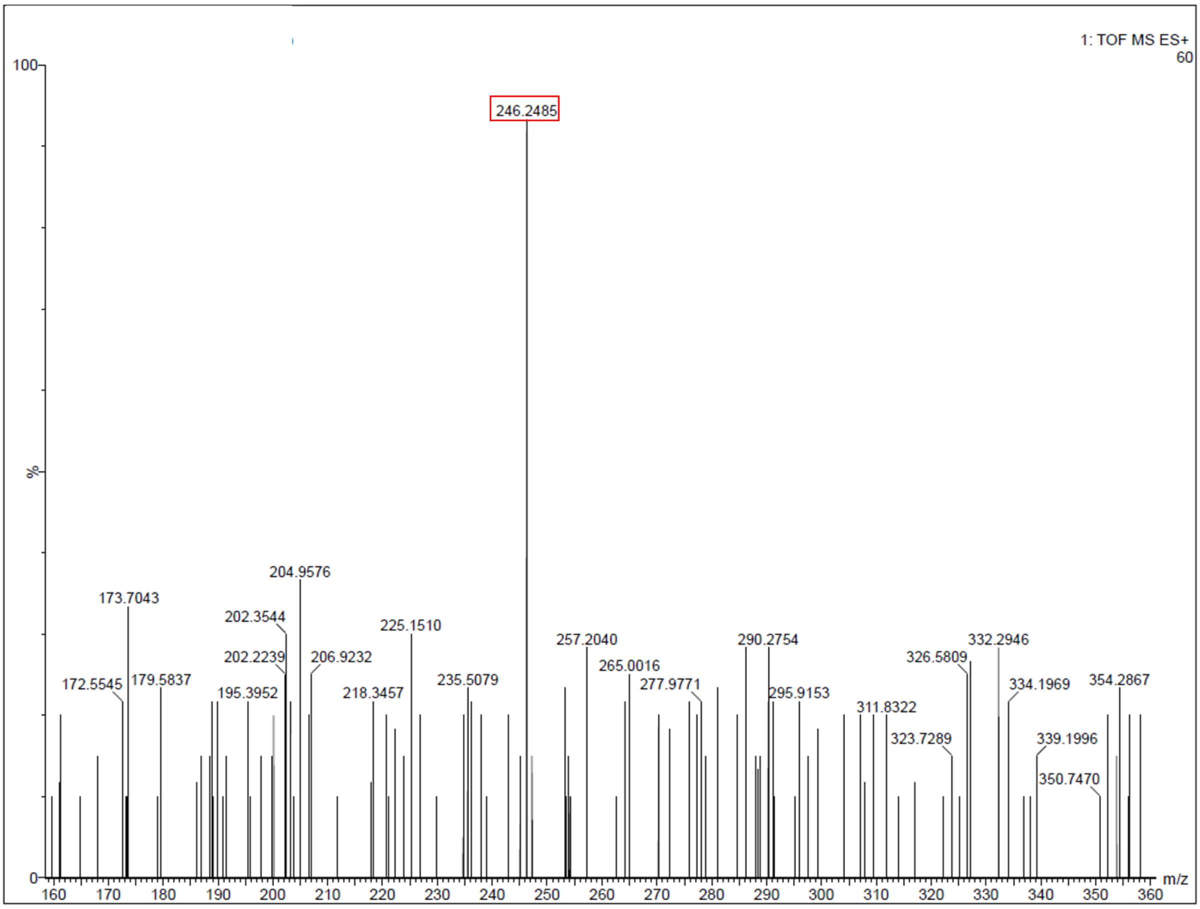


**Figure S8.** Mass spectra of glycine residue obtained after impact. Identified peptide peak ($M^+$) is shown in red box corresponds to 246.2485 matches with calculated value 246.207 (peptide sequence-GGGG)

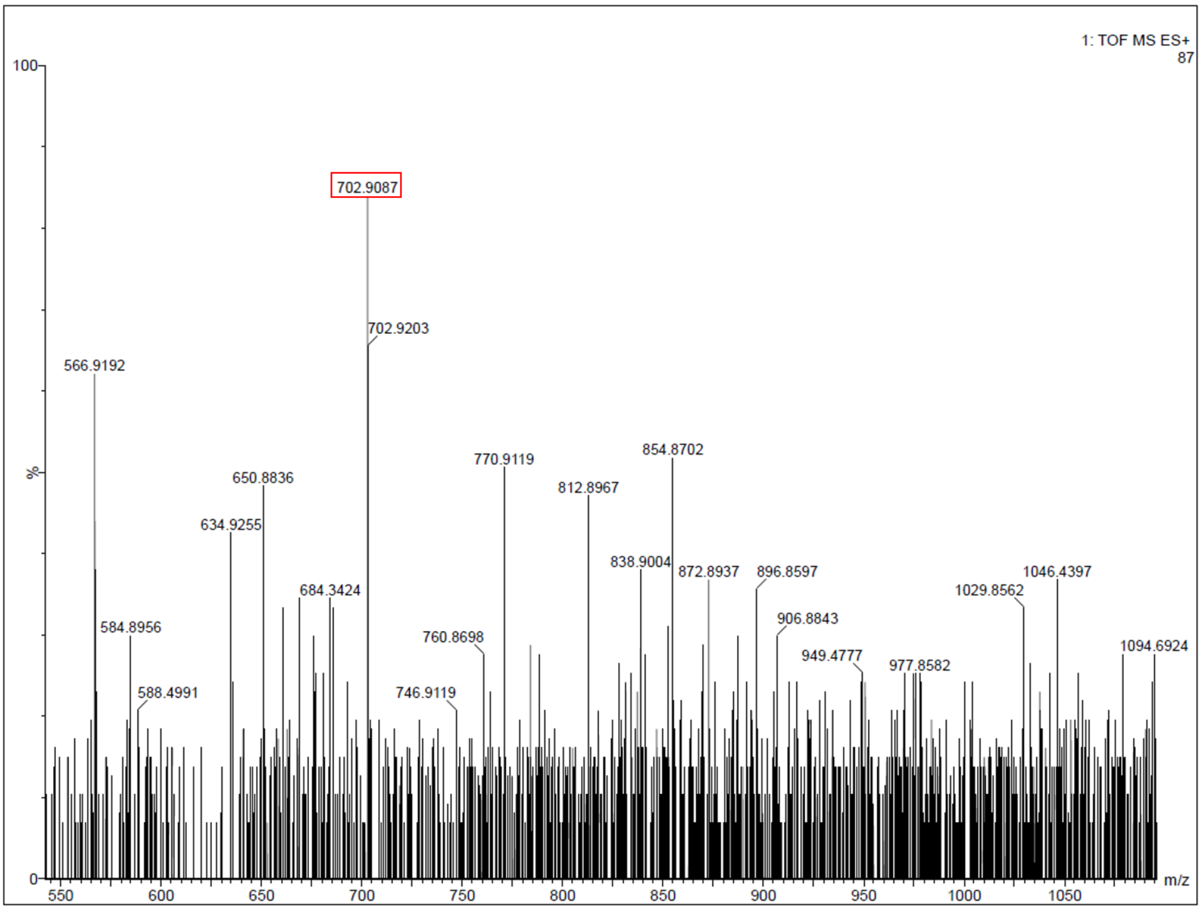


**Figure S9.** Mass spectra of glycine residue obtained after impact. Identified peptide peak ($M^+$) is shown in red box corresponds to 702.9087 matches with calculated value 702.621 (peptide sequence of 12 G's)

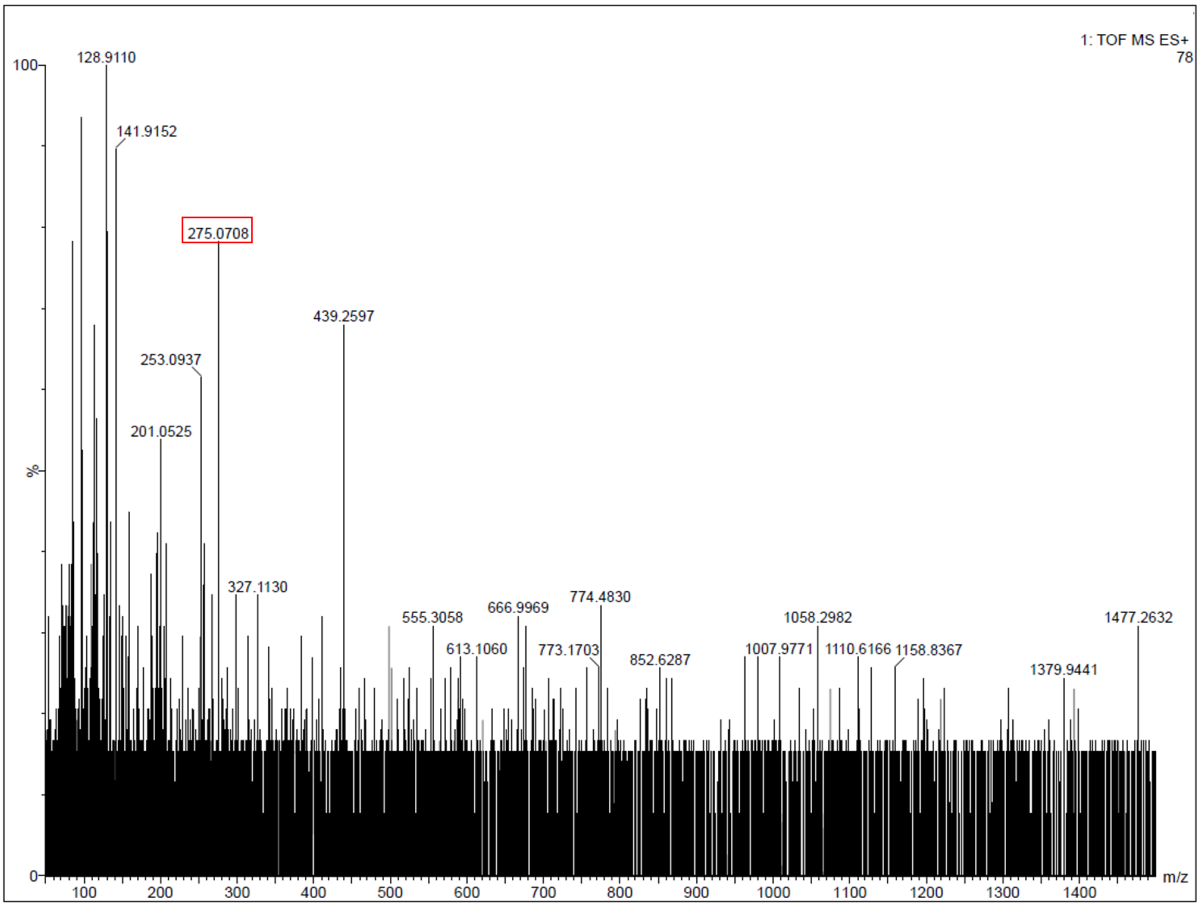


**Figure S10.** Mass spectra of glutamine residue obtained after impact. Identified peptide peak ($MH^+$) is shown in red box corresponds to 275.0708 matches with calculated value 274.261 (peptide sequence - 2 Q's)

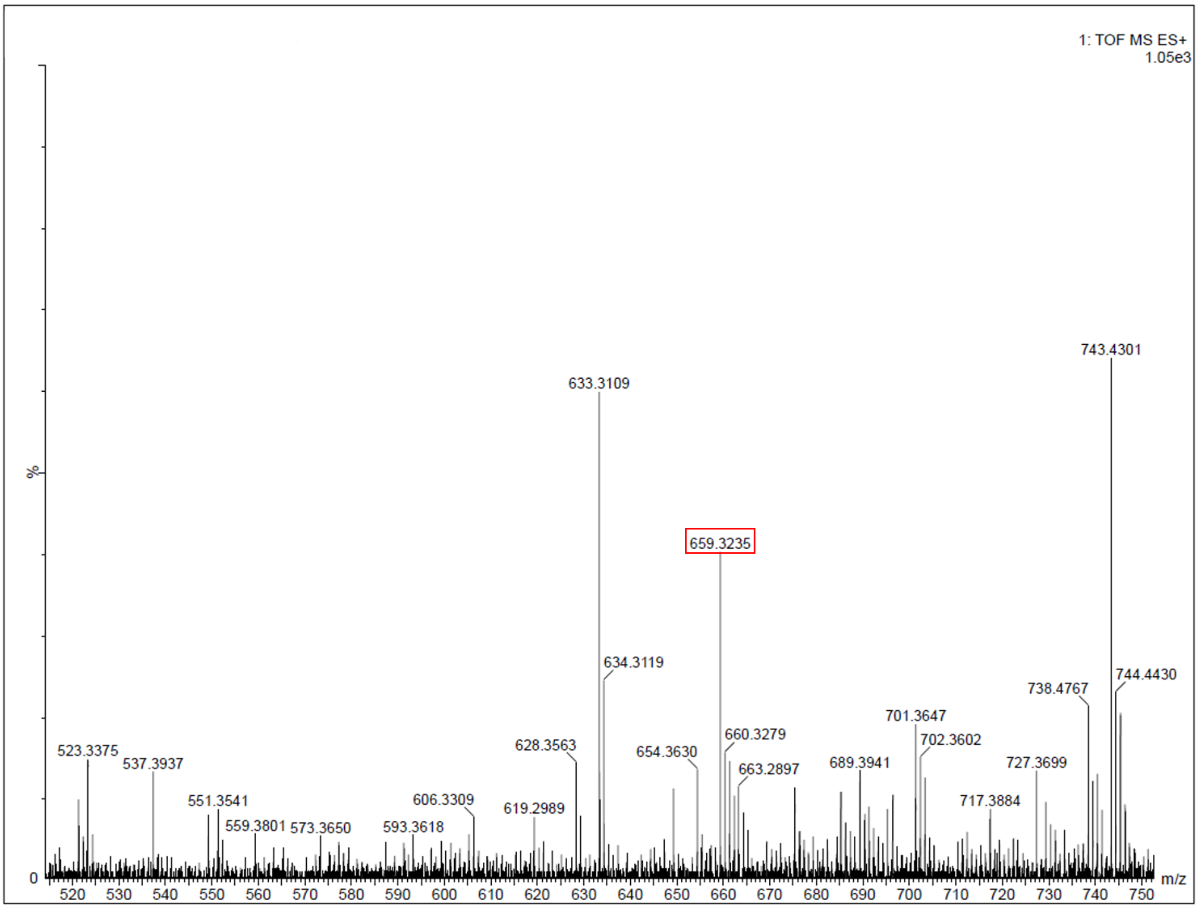


**Figure S11.** Mass spectra of glutamine residue obtained after impact. Identified peptide peak ($MH^+$) is shown in red box corresponds to 659.3235 matches with calculated value 658.652 (peptide sequence – 5 Q's)

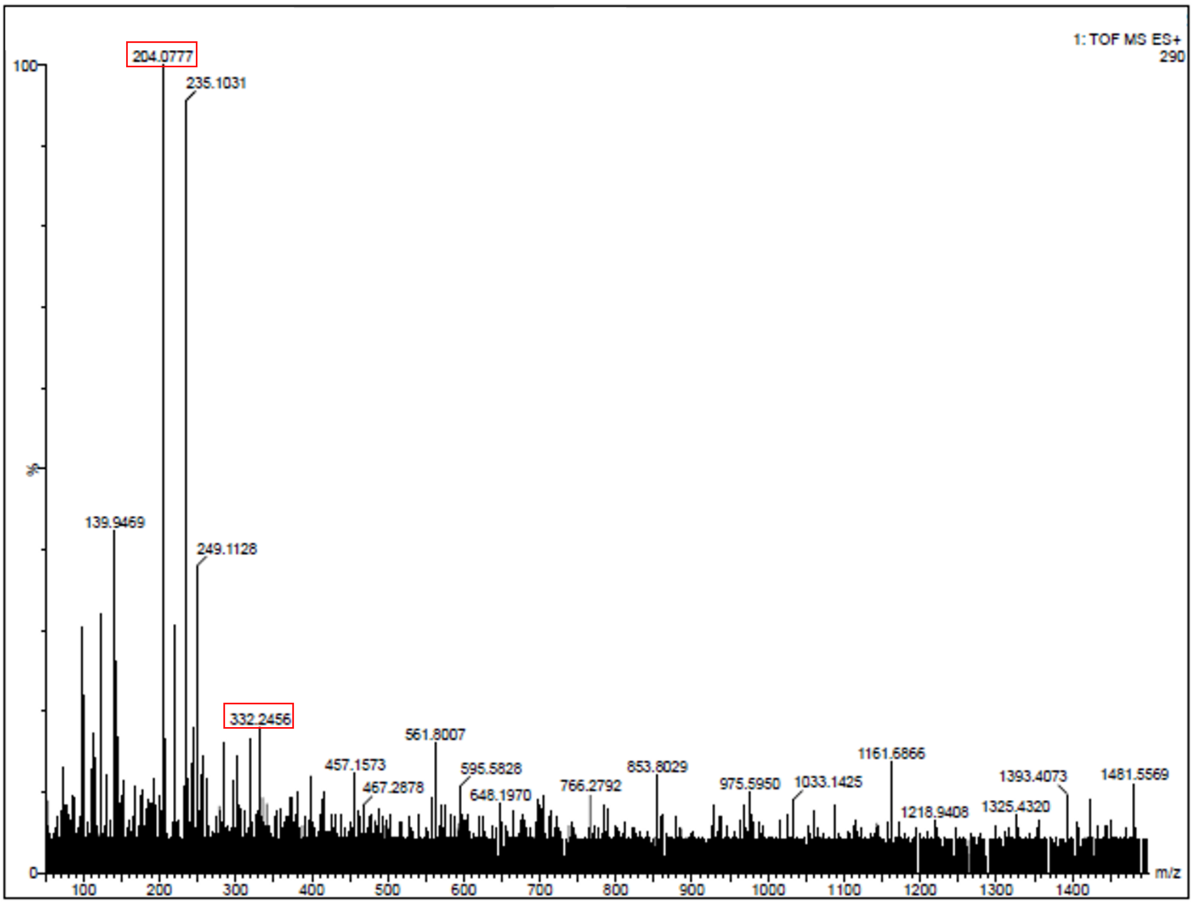


**Figure S12.** Mass spectra of glycine-glutamine residue obtained after impact. Identified peptide peak ($MH^+$) is shown in red box corresponds to 204.077 and 332.2456 matches with calculated value 203.182 (peptide sequence – 1 G and 1 Q) and 331.312 (peptide sequence – 1 G and 2 Q's) respectively.

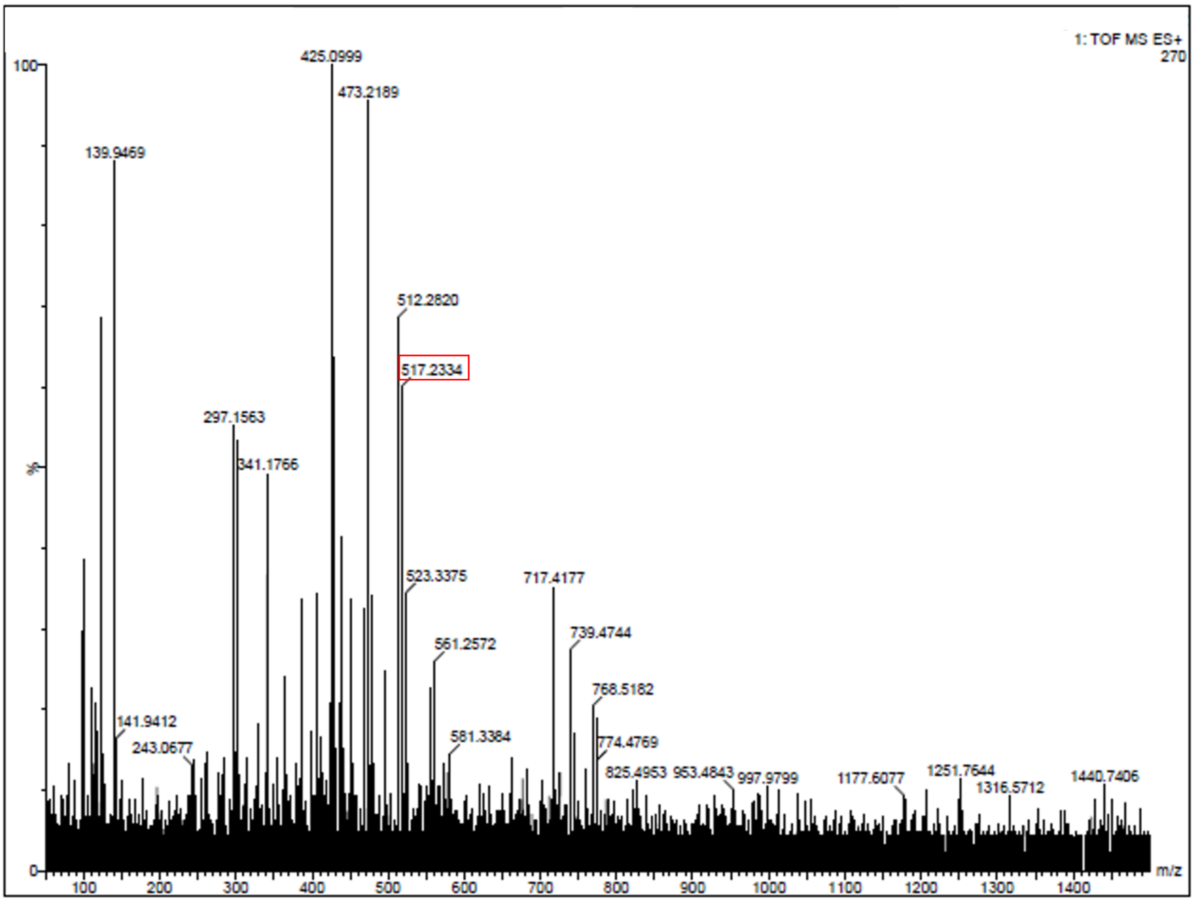


**Figure S13.** Mass spectra of glycine-glutamine residue obtained after impact. Identified peptide peak ($MH^+$) is shown in red box corresponds to 517.2334 matches with calculated value 516.495 (peptide sequence – 2 G's and 3 Q's).

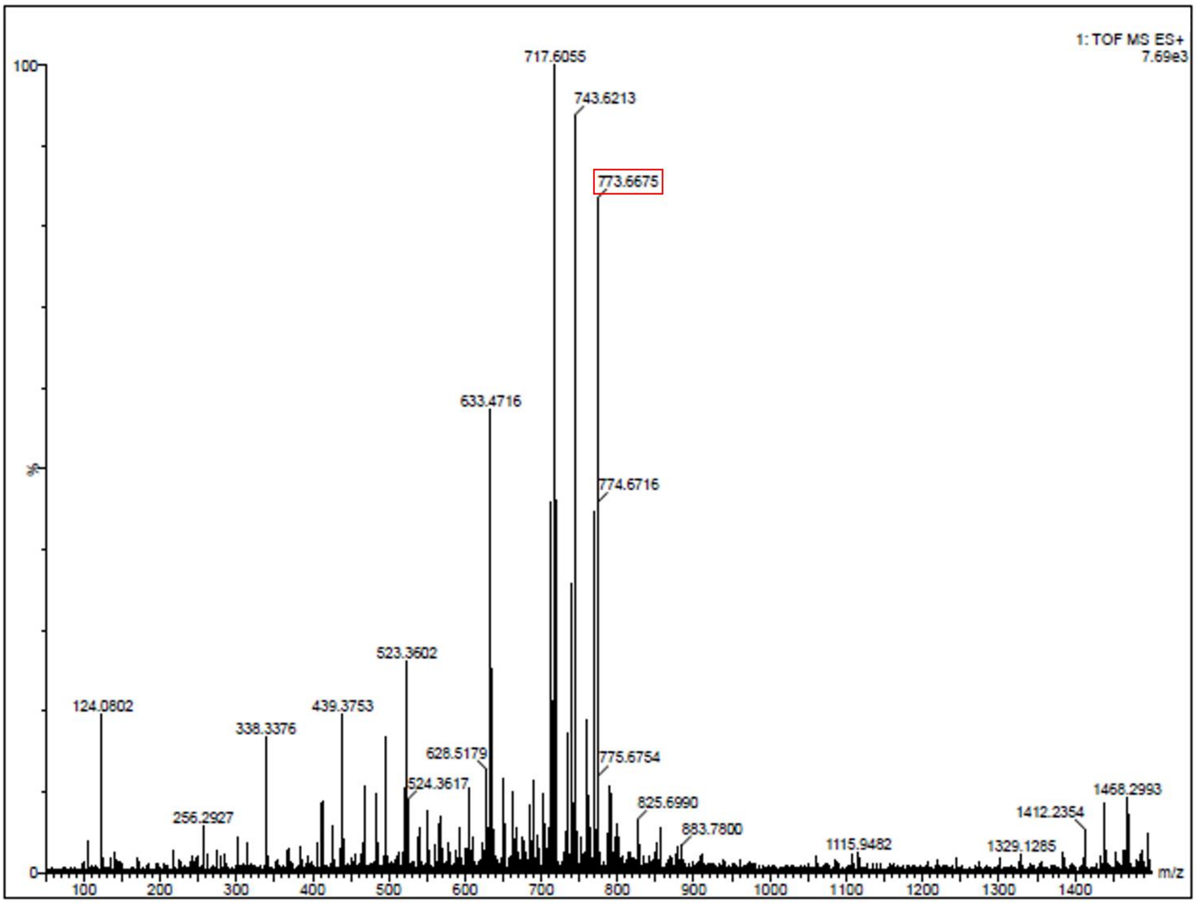


**Figure S14.** Mass spectra of glycine-glutamine residue obtained after impact. Identified peptide peak ($MH^+$) is shown in red box corresponds to 773.6675 matches with calculated value 772.756 (peptide sequence – 2 G's and 5 Q's)
